# Bolt Anchorage Defect Identification Based on Ultrasonic Guided Wave and Deep Learning

**DOI:** 10.3390/s25206431

**Published:** 2025-10-17

**Authors:** Hui Xing, Weiguo Di, Xiaoyun Sun, Mingming Wang, Chaobo Li

**Affiliations:** 1 School of Electrical and Electronic Engineering, Shijiazhuang Tiedao University, Shijiazhuang 050043, China; xinghui@stdu.edu.cn (H.X.); weiguodi@126.com (W.D.); wangmm@stdu.edu.cn (M.W.); 2 School of Electronic and Control Engineering, North China Institute of Aerospace Engineering, Langfang 065000, China; 3 Hebei Academy of Emergency Management Sciences, Shijiazhuang 050031, China; 18630121072@126.com

**Keywords:** anchorage recognition, non-destructive testing, ultrasonic guided wave, continuous wavelet transform, deep learning

## Abstract

As a critical supporting component in geotechnical engineering structures such as bridges, tunnels, and highways, the anchorage quality of bolts directly impacts their structural safety. The ultrasonic guided wave method is a popular method for the non-destructive testing of anchorage quality. However, noise from complex field environments, modal mixing caused by anchoring interface reflections, and dispersion effects make it challenging to directly extract defect features from guided wave signals in the time or frequency domains. To address these challenges, this study proposes a solution based on the combination of the guided wave time–frequency spectrum and the gated attention residual network (GA-ResNet). The GA-ResNet introduces a gating mechanism to balance spatial attention and channel attention, and it is used for anchoring model type recognition. Experiments were conducted on four types of anchorage models, and the time–frequency spectrum was selected to be the input feature. The results demonstrate that the GA-ResNet can effectively predict the anchorage bolt defect type and prevent potential safety accidents.

## 1. Introduction

Anchorage bolts are widely used in mining engineering, underground engineering, and slope support engineering [1]. After being bolted into structures, anchorage bolts bear tensile and shear loads through interfacial bonding with structures such as concrete and rock mass to both prevent their movement and deformation and maintain their long-term stability. Influenced by material properties, on-site construction conditions, and the ability of skilled workers, anchorage systems may have defects such as insufficient grouting and local debonding during the construction process. However, anchoring constructions are highly concealed, and their problems cannot be directly discovered. Such defects significantly weaken the bearing capacity of anchoring systems. In severe cases, they may lead to major engineering safety incidents, such as surrounding rock collapse and structural instability. Therefore, achieving an accurate detection of bolt anchoring quality and defect identification is crucial for mitigating engineering risks and ensuring operation safety.

Traditional anchorage quality testing mainly relies on the pull-out test [2,3,4,5]. Although this method can directly obtain anchorage force, it is a destructive, high-cost, and low-efficiency method which struggles to meet the large-scale testing needs of engineering sites. Non-destructive testing (NDT) technology, with its advantages such as high efficiency, has become an important approach for bolt anchoring quality evaluation. To date, researchers have developed a variety of NDT technologies, including the stress wave method, ultrasonic guided wave method, and magnetostrictive guided wave method [6,7,8]. Beard et al. [6] applied ultrasonic guided waves for bolt anchorage testing, confirming that this technology can effectively identify bolt length and defects such as necking and resin encapsulation failure. He et al. [7] utilized low-frequency ultrasonic longitudinal guided waves and achieved quantitative detection of the debonding length by analyzing the time difference between the reflected echoes from the bottom end of the bolt and the upper interface of the anchoring section. Lei M. et al. [8] proposed an automatic extraction algorithm based on the principle of stress wave propagation, realizing the accurate measurement of the length of in-service bolts. These studies indicate that there is a significant correlation between the phase distribution, amplitude variation, energy attenuation, and wave velocity characteristics of ultrasonic guided waves and anchoring defects. This correlation provides a theoretical basis for defect identification. However, the existing analyses of the above research mainly focus on the guided waves themselves, and determine the anchorage length or defect position by picking the arrival time of the original signal without considering the influence of noise and mode mixing.

In practical engineering, ultrasonic guided waves are contaminated with substantial environmental noise. The reflection of guided waves at multiple interfaces leads to a prominent problem of mode mixing; meanwhile, the dispersion effect of different frequency components further increases the difficulty of signal analysis. These challenges significantly increase the difficulty of extracting defect features directly from time-domain or frequency-domain signals, seriously restricting the detection accuracy.

To address the aforementioned issues, signal processing methods such as Fourier transform [9], wavelet transform [9,10,11,12,13,14,15], and mode decomposition [16,17,18,19,20,21] have been introduced. Wavelet transform is widely used in ultrasonic guided wave signal processing due to its excellent time–frequency localization characteristics. Jianhui Y. et al. [11] utilized wavelet filter to achieve an accurate identification of the free segment length, grouting length, and defect location of hollow grouted bolts. Li et al. [12] confirmed that this method can effectively detect grouting defects with specific widths by comparing the wavelet packet energy values of specimens with and without grouting defects. Yi T. et al. [13] successfully identified the length of the anchoring section, defect length, and specific defect locations with different wavelet filters. J.S. Lee et al. [14] investigated the unbonded length of a rock bolt using the built-in microphone of a smartphone, used wavelet transform for time–frequency analysis, and identified that the predominant frequency was the resonant effect of the reverberant sound waves. Liu et al. [15] used the fractal box dimension of wavelet packet decomposition as the main feature to predict rock bolt debonding status. In addition, mode decomposition methods have also been applied in guided wave signal analysis. Guo et al. [19] employed improved complete ensemble empirical mode decomposition (ICEEMD) combined with wavelet soft threshold denoising technology to successfully identify reflection signals. Li et al. [20,21] decomposed the bolt stress waves using variational mode decomposition (VMD), extracted the bottom-reflected signals, and further calculated the anchoring length and defect length. These signal processing methods have somewhat improved signal quality, but still rely on manual experience for feature parameter selection and struggle to address the issues of strong noise and mode superposition in complex environments.

In recent years, neural networks, with their powerful adaptive learning capabilities, have provided a new approach for bolt anchorage quality detection based on guided waves. This technology can automatically learn the guided wave features of different defect types, reduce manual intervention, and improve identification accuracy. Sun et al. [22] extracted guided wave features through wavelet packet energy analysis and used them as input vectors for an improved probabilistic neural network, achieving classification and identification of anchoring defects. Di et al. [23] proposed an improved chicken swarm optimization algorithm to optimize the Elman neural network, with the principal components of the frequency response function as inputs, significantly enhancing defect identification accuracy. Yu et al. [24,25] constructed a three-dimensional bolt numerical model to simulate the propagation process of longitudinal waves, and used a neural network to establish the mapping relationship between the longitudinal wave velocity and rock properties. Han et al. [26] prepared corroded bolts through electrochemical experiments, and combined guided wave data with a multi-scale convolutional neural network to achieve quantitative diagnosis of corrosion degree. Most of the studies utilize time-domain features of the original signal for anchor type prediction without considering frequency-domain information. Further research is needed to detect anchorage quality under more complex conditions based on richer guided wave features.

This paper proposes a bolt anchorage defect prediction method based on ultrasonic guided wave time–frequency features and the GA-ResNet. The time–frequency spectrum couples abundant time- and frequency-domain information and can provide richer features for deep learning models. The basic module of the GA-ResNet model is the gated attention residual block (GARB), which employs a gated mechanism to adjust the weights of channel attention and spatial attention and extract features effectively. After proposing this method, we achieved high-precision prediction of anchorage quality with GA-ResNet.

## 2. The Relevant and Proposed Method

### 2.1. Continuous Wavelet Transform

The ultrasonic guided wave signals detected from bolts are typical non-stationary signals. Their time-domain waveforms are disturbed by noise and mode superposition, while the spectral information is often affected by the truncation window. Time–frequency analysis can present both the time-domain and frequency-domain characteristics of signals simultaneously, providing richer feature information for defect identification. This paper uses CWT to convert guided wave signals into time–frequency diagrams, realizing the visualization and enhancement of defect features.

The CWT of an input signal *x*(*t*) is mathematically defined by the following:

(1) $${\mathrm{CWT}}_{x}(a,b)=\int x(t){\psi^{*}}_{a,b}(t)dt=\frac{1}{\sqrt{a}}\int x(t)\psi^{*}\left[\frac{t-b}{a}\right]dt$$

where a is the scaling factor, b is the translation factor, and ${\psi^{*}}_{a,b}(t)$ is the wavelet base. A complex Morlet wavelet is employed in this paper, and its transformation results can preserve both the amplitude and phase information of guided wave signals, clearly revealing the energy distribution changes caused by defects. Here, we only use the absolute value of wavelet coefficients because of its advantage of rich time–frequency spectrum information for deep learning models. The complex Morlet wavelet is expressed as follows:

(2) $$\psi (t)=\frac{1}{\sqrt{\pi f_{B}}}e^{j2\pi f_{c}t}e^{-t^{2}/f_{B}}$$

where *f*_c_ is the center frequency and *f*_B_ is the bandwidth. The central frequency *f*_c_ and bandwidth *f*_B_ are both 1 Hz.

### 2.2. GA-ResNet Model

The residual network (ResNet) model was proposed by He et al. [27]. By introducing residual blocks and skip connections, it effectively addresses the gradient vanishing problem in the deep neural network training process, significantly improving the model’s feature extraction capability. Considering the scale and complexity of the dataset used in this study, the GA-ResNet is designed based on the ResNet-18 architecture. By incorporating the attention mechanism into the residual blocks, the model’s ability to focus on key defect features in time–frequency diagrams is enhanced.

#### 2.2.1. Model Overall Architecture

**Figure 1:**
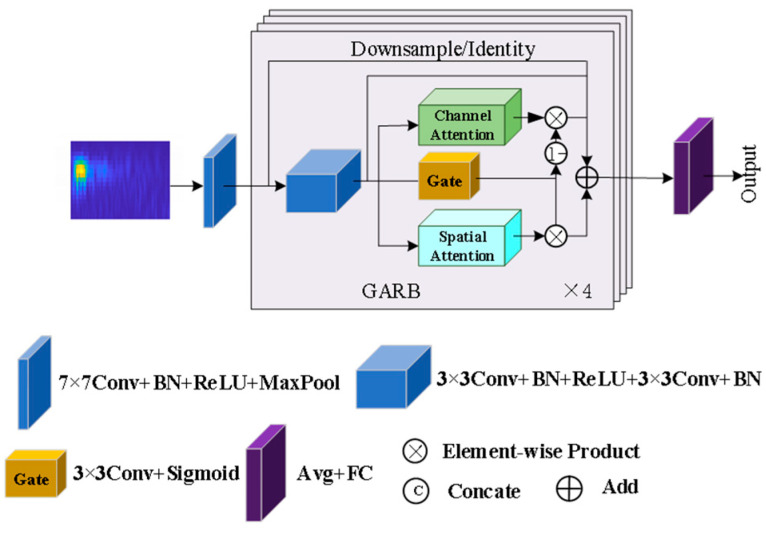
Architecture of the GA-ResNet.

The GA-ResNet model mainly consists of a shallow feature extraction layer and four cascaded GARBs, as shown in Figure 1. The shallow feature extraction layer uses a 7 × 7 convolution kernel to perform convolution operations on the input time–frequency diagrams, thereby extracting the initial global features. The GARB serves as the basic module of the model, incorporating the CA, SA, and GA mechanisms to realize the dynamic enhancement of key features. After passing through the four cascaded GARBs, the feature maps complete the identification of anchorage defect types through the global average pooling layer and the fully connected layer.

#### 2.2.2. Gated Attention Residual Block

**Figure 2:**
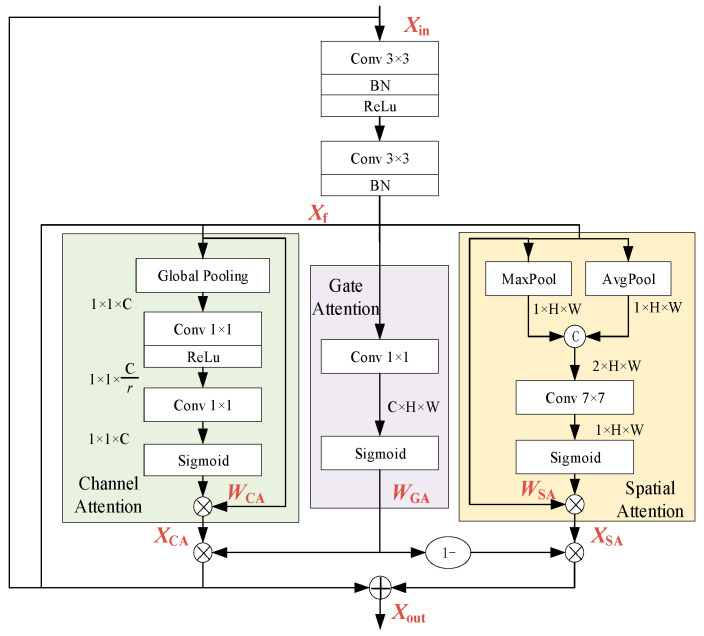
GARB.

The GARB proposed in this paper utilizes spatial attention and channel attention [28] while introducing a gating mechanism to balance the weights between them, as shown in Figure 2.

First, the input features *X_in_* of each GARB pass through a two-layer convolution layer to obtain the initially extracted feature map *X_f_*. This process is identical to the structure of a general residual block, and it is expressed as follows:

(3) $$X_{f}=\mathrm{BN}({\mathrm{Conv}}_{3\times 3}(\mathrm{ReLU}(\mathrm{BN}({\mathrm{Conv}}_{3\times 3}(X_{\mathrm{in}})))))$$

where Conv_3×3_(·) denotes the 3 × 3 convolution operation, BN stands for batch normalization, and ReLU is the activation function.

The attention module consists of three branches: channel attention, spatial attention and gated attention. Channel attention is good at filtering effective channels of the feature maps, spatial attention has a great ability to focus on key areas, and gate attention assigns weights to different channels and positions to avoid interference from irrelevant information.

The channel attention branch obtains the channel weight vector *W_CA_* through average pooling over the spatial dimension, a multi-layer perceptron (MLP), and an activation function, which enhances the channel features related to defects in the feature map *X_f_*. The output feature *X_CA_* is calculated as follows:

(4) $$W_{\mathrm{CA}}=\sigma (\mathrm{MLP}(\mathrm{Avg}(X_{f})))$$

(5) $$X_{\mathrm{CA}}=W_{\mathrm{CA}}⊗X_{f}$$

where Avg(·) denotes global average pooling, σ(·) denotes the sigmoid activation function, and ⊗ denotes the element-wise product.

After performing average pooling and max pooling along the channel dimension, the spatial attention branch obtains the spatial weight vector *W_SA_* through convolution and activation. This process focuses on the spatial regions related to defects while reducing the impact of noise, and it is expressed as follows:

(6) $$W_{\mathrm{SA}}=\sigma ({\mathrm{Conv}}_{7\times 7}([\mathrm{Avg}(X_{f});\mathrm{MAX}(X_{f})]))$$

(7) $$X_{\mathrm{SA}}=W_{\mathrm{SA}}⊗X_{f}$$

where Conv_7×7_(·) denotes a 7 × 7 convolution layer and [·;·] denotes the concatenation operation.

The gated attention obtains gating parameters through a convolution layer and an activation function, which adaptively adjusts the fusion weights of channel attention and spatial attention, thereby further optimizing the feature selection effect, and it is expressed as follows:

(8) $$W_{\mathrm{GA}}=\sigma ({\mathrm{Conv}}_{1\times 1}(X_{f}))$$

To accommodate the size of input feature *X_in_*, the first GARB uses an identity connection, while the others employ downsampling connections, and it is expressed as follows:

(9) $$X_{\mathrm{out}}=X_{\mathrm{CA}}⊗W_{\mathrm{GA}}+X_{\mathrm{SA}}⊗(1-W_{\mathrm{GA}})+X_{f}+F(X_{\mathrm{in}})$$

where F(·) denotes an identity connection or a downsampling connection.

## 3. Experiments

### 3.1. Ultrasonic Guided Wave Test for Bolt Anchorage System

#### 3.1.1. Test Design and Implementation

**Figure 3:**
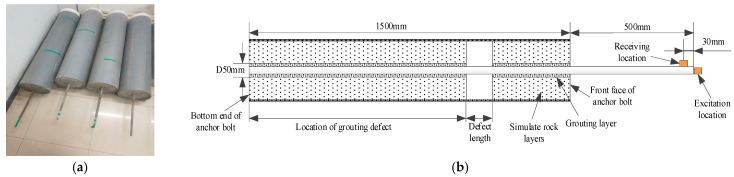
Bolt anchorage model structure. (**a**) Actual models (from right to left: Models 1, 2, 4, and 3), the green lines on the PVC pipe indicate the locations of the defects; (**b**) model section structure.

The bolt anchorage models and their internal structures are shown in Figure 3. The models are made of steel bars, concrete, and PVC pipes. The steel bar and PVC pipe have lengths of 2000 mm and 1500 mm, and diameters of 20 mm and 300 mm, respectively. The model preparation is divided into two processes. First, we placed a 50 mm diameter pipe into the center of the PVC pipe, and we filled the space between them with concrete material 1. During the concrete solidification process, we extracted the 50 mm pipe. Second, the steel bar was fixed in the center of the 50 mm hole after concrete 1 had solidified. Then, we used concrete material 2 to fill the hole, and it was also filled with foam material to simulate defects. Four types of anchorage models were prepared for the test, and the defect location parameters of each model are listed in Table 1. The composition ratios of concrete materials 1 and 2 are shown in Table 2.

**Figure 4:**
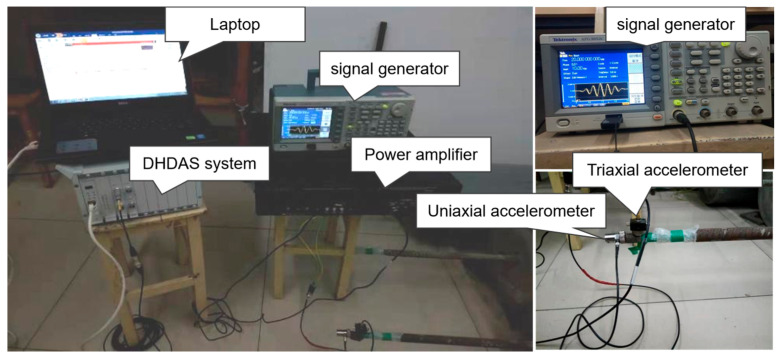
Actual signal acquisition.

The non-destructive testing system for bolt anchorage is shown in Figure 4, which mainly consists of an excitation module, a signal-receiving module, and a data acquisition module. During the test, a Tektronix AFG3052C signal generator (From China Tektronix Technology Co., Ltd., Shanghai, China) was employed to generate a five-period sine wave with an excitation frequency of 20 kHz as the excitation signal. To increase the signal amplitude and reduce noise, an AE Techron 7224 power amplifier (AE Techron, Inc., Elkhart, IN, USA) was employed to amplify the excitation signal. The amplified signal was applied to the end face of the free bolt via a uniaxial accelerometer, generating longitudinal waves that propagate axially along the bolt. A triaxial accelerometer was used to receive the reflected wave signals at the side of the end face, and the DHDAS dynamic signal acquisition system was employed to convert the received reflected wave signals and transmit them to a computer.

#### 3.1.2. Ultrasonic Guided Wave Signal Analysis

In actual testing process, multiple factors may affect the test results, such as sensor position, coupling conditions, environmental noise, and the selection of the guided wave starting point. These factors can introduce various distortions, increasing the complexity of guided waves.

**Figure 5:**
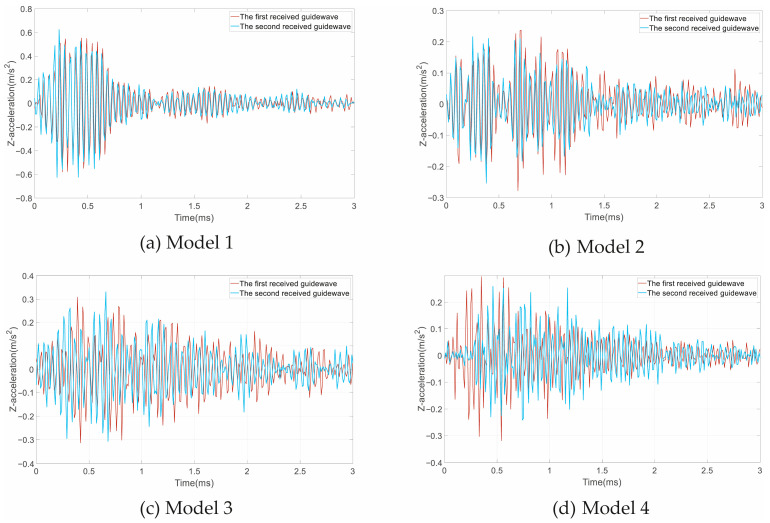
Ultrasonic guided waves of the four models: (**a**) Model 1; (**b**) Model 2; (**c**) Model 3; (**d**) Model 4.

Two 3 ms waveforms collected at different times were extracted from the acceleration signals, as shown in Figure 5. The arrival times and velocities of waves are shown in Table 3. The wave velocities of the free and anchorage sections of the anchorage bolts can be calculated by *v* = *L*/Δ*t*, where *L* is the length of free bolt or anchorage section and Δ*t* is the propagation time of guide wave.

In Figure 5a, the two received waves have a very small difference because of the of selection starting time. We select the middle peak time of the wave packets as the reflection wave time after comparing the two waves. Using the arrival times, we can calculate that the guide wave speed in the free bolt is 5288.0 m/s, and the speed in the anchorage section has a very small difference of 51.1 m/s. In Figure 5b, the peak and trough time of the two guided waves are basically consistent, but there are significant deviations in amplitude. Due to the staircase distortions, the arrival times have a significant error. The calculated velocity of Model 2’s second received guide wave is as high as 5867.7 m/s. In Figure 5c,d, influenced by the initial truncation time, the peaks and troughs of the two guided waves do not overlap at all; therefore, we can hardly determine the middle peaks of wave packets. At the same time, more staircase distortions appear due to the defects and model mixing. In this situation, we mainly determine the arriving times according to the maximum peaks and personal experience, meaning that there are large differences in the velocities.

**Figure 6:**
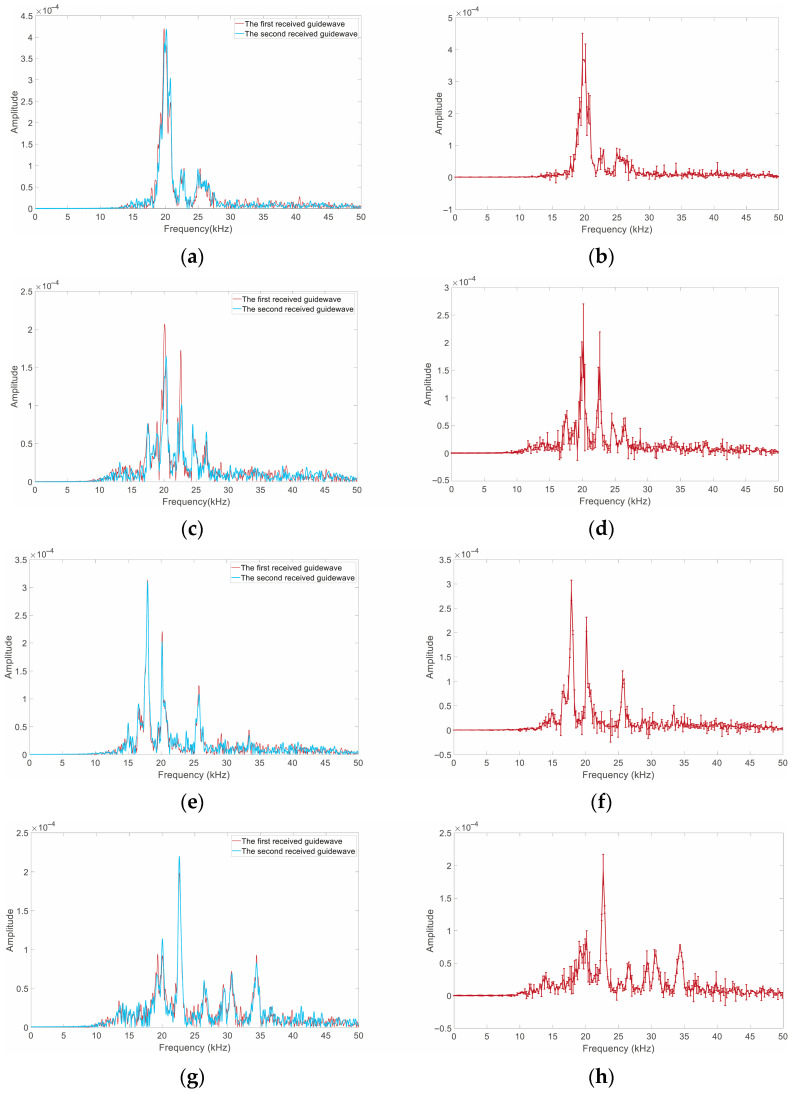
Ultrasonic guided wave spectrum of the four models: (**a**) frequencies of Model 1; (**b**) error bar for frequencies of Model 1; (**c**) frequencies of Model 2; (**d**) error bar for frequencies of Model 2; (**e**) frequencies of Model 3; (**f**) error bar for frequencies of Model 3; (**g**) frequencies of Model 4; (**h**) error bar for frequencies of model 4.

Figure 6 shows the frequency spectrum and its error bar of the guided waves from four bolt models after fast Fourier transform (FFT). The spectral distributions of Model 1 are relatively concentrated, and there is only a small deviation between two spectrum curves. Their dominant frequencies are 19.77 kHz and 20.13 kHz, respectively. Model 2’s (single small defect) spectrums appear to be decentralization. The dominant frequencies are 20.11 kHz and 20.32 kHz, respectively, but the difference in amplitude is quite large. The dominant frequencies of Models 1 and 2 are very close, perhaps due to their relatively small defects. The dominant and sub-dominant frequencies of Model 3 are 17.91 kHz and 20.16 kHz (the first received guide wave), or 17.95 kH and 20.16 kHz (the second received guide wave). The dominant frequencies shift to the left compared to those in Models 1 and 2; however, the sub-dominant frequencies are almost the same as the dominant frequencies of Models 1 and 2. Compared to Models 1 and 2, the dominant frequencies of Model 4 shifted to the right at 22.61 kHz and 22.63 kHz, respectively. The first received wave has three sub-dominant frequencies with very close amplitudes, located at 19.32 kHz, 20.06 kHz, and 34.41 kHz, respectively. Meanwhile, the second received wave only has one, at 20.05 kHz. This difference may be due to the different truncation times at the initial moment.

The spectrum differences in the same model mainly stem from the non-stationary characteristics of ultrasonic guided waves. The initial truncation time has a smaller impact on the spectral distribution than the time-domain waveform, but it still can be seen that making a reliable distinction between anchorage defect types cannot be achieved by frequency-domain analysis alone.

**Figure 7:**
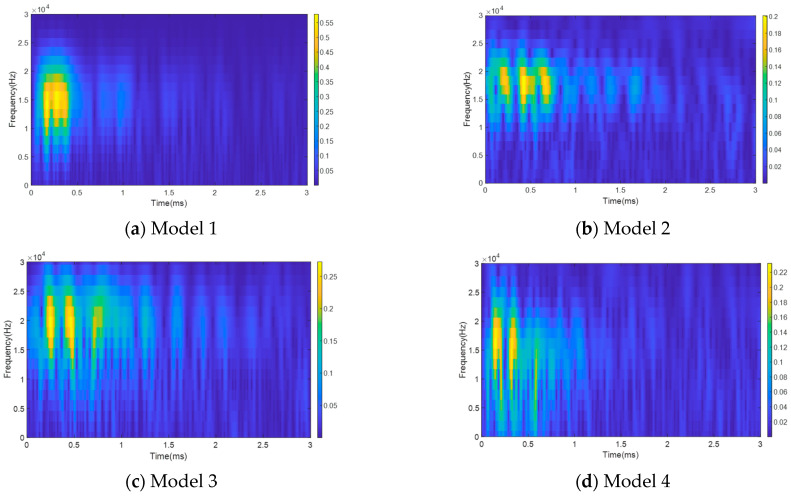
Wavelet time–frequency diagrams of ultrasonic guided waves for the four models: (**a**) Model 1; (**b**) Model 2; (**c**) Model 3; (**d**) Model 4.

To address the limitations of time-domain and frequency-domain analyses, this study employs the CWT on all acquired guided waves to achieve the visualization of time–frequency coupled features. Figure 7 presents the wavelet time–frequency diagrams of the guided wave signals for the four models, where warm and cool colors represent the energy values of different frequency components. The time–frequency diagram of Model 1 (complete anchorage) shows highly concentrated energy, primarily distributed within the 0–0.5 ms time range and the 10–20 kHz frequency range, indicating that the wave propagation was undisturbed by defects. In contrast, the energy distribution for Model 2 (a single small defect) becomes scattered, with the time range expanding to 0–0.8 ms, which is primarily attributed to wave reflections at the defect interface. As the number or size of the defects increases, the energy dispersion in Models 3 (two small defects) and 4 (a single large defect) intensifies considerably, exhibiting more severe energy scattering. This phenomenon is associated with the continuous reflection and transmission of guided waves at the grouting interfaces of the defects.

### 3.2. Dataset Construction

**Figure 8:**
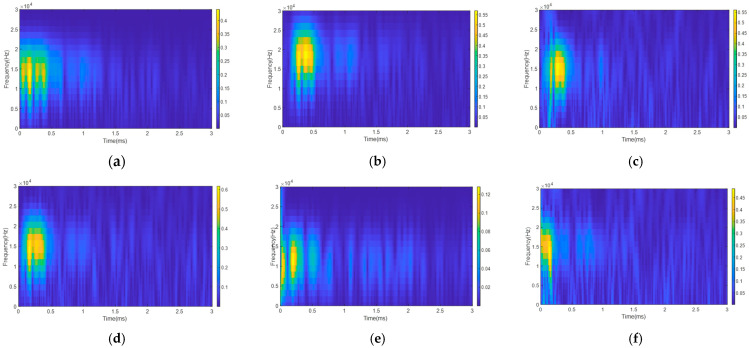
Augmented time–frequency spectrum of Model 1: (**a**) VMD denoising; (**b**) different initial moments; (**c**) salt-and-pepper noise; (**d**) Gaussian noise; (**e**) different initial moments and VMD denoising; (**f**) different initial moments and salt-and-pepper noise.

A total of 150 laboratory tests were conducted on each anchorage model, resulting in a total of 600 datasets. To increase the diversity of guided waves, we processed the original signals by several methods, such as denoising via variational mode decomposition (VMD), intercepting at different initial moments, and adding salt-and-pepper noise and Gaussian noise. A number of time–frequency spectrums of the processed signals are shown in Figure 8. After the augmentation, we obtained a total of 5800 spectrum images, comprising 1450 images for each model. We randomly selected 40 and 60 images from each model dataset as test and validation sets, and used the remaining 1350 images as the training set.

## 4. Results and Discussion

A computer equipped with an NVIDIA GeForce RTX 4060 Laptop GPU was used for the GA-ResNet model. The programming environment included PyTorch 1.8.1 and CUDA 11.1. For model training, the batch_size was set to 20 and the number of epochs was 20, with a validation test triggered every 20 batches. The model adopted the Stochastic Gradient Descent with Momentum (SGDm) optimizer, where the momentum parameter was set to 0.9 and the initial learning rate was 0.001. The learning rate decay factor was 0.1, and the decay interval was five epochs.

### 4.1. Comparison of Different Models

**Figure 9:**
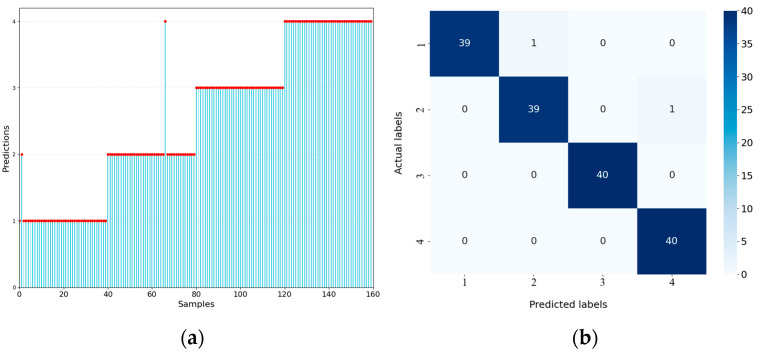
Predictions of the GA-ResNet: (**a**) prediction results (the red dots) for each sample; (**b**) confusion matrix.

The prediction results of the GA-ResNet are presented in Figure 9. The prediction accuracies of Model 3 and Model 4 all reach 100%. Sample 2 of Model 1 and Sample 67 of Model 2, both of which were signals with high salt-and-pepper noise, were misclassified. These results indicate that extreme noise can lead to recognition failure.

**Figure 10:**
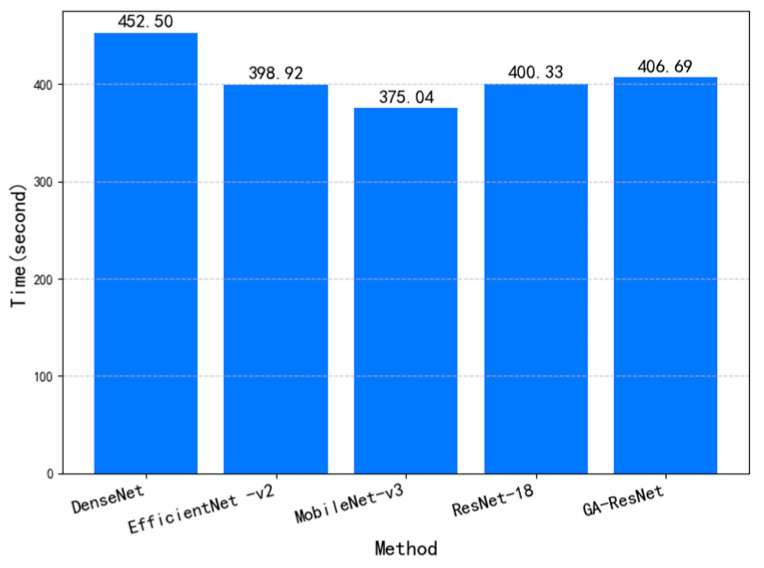
Training time per epoch of different methods.

To verify the superiority of the proposed algorithm, ResNet-18, DenseNet [29], MobileNetV3-S [30], and EfficientNetV2-S [31] were selected as comparative models, and all input samples were resized to 224 × 224 × 3. Under the same training parameter settings, the training time per epoch for each model is shown in Figure 10. DenseNet required the longest training time, while MobileNetV3-S needed the least. The training time of the proposed GA-ResNet model was slightly higher than that of ResNet-18. This increase is due to the introduction of the attention mechanism in the GA-ResNet, which adds a modest computational overhead. Nevertheless, the computational cost remains within an acceptable range without significantly sacrificing training efficiency.

**Figure 11:**
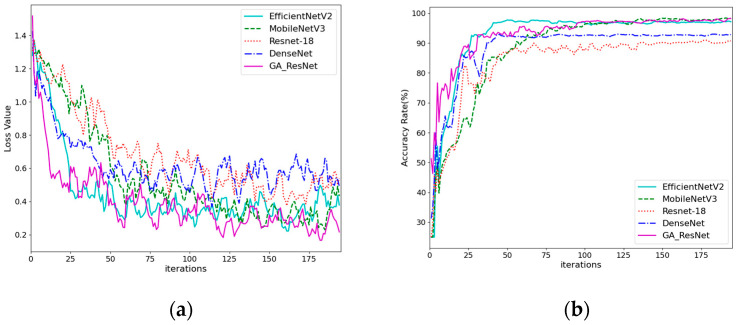
Training loss and validation accuracy curves of different models: (**a**) train losses; (**b**) validation accuracy.

The training loss function curves and validation accuracy curves of different models are shown in Figure 11. During training, the GA-ResNet exhibits the fastest decline in loss, and achieves a minimum loss of approximately 0.04. This indicates that the model has a high gradient descent efficiency. EfficientNetV2-S shows a slightly slower decline in loss, with slightly larger fluctuations. The loss of MobileNetV3-S decreases slowly in the early stage, but after 120 iterations, it approximates that of EfficientNetV2-S. The DenseNet loss decreases rapidly in the early stage, but the final convergence effect is the worst, and ResNet-18 performs poorly throughout the entire training process. The GA-ResNet demonstrated the fastest rise in validation accuracy, and EfficientNetV2-S exhibits a slightly slower rise at first, but it is the fastest to reach an accuracy rate of 98.33%. Similarly to training loss, the validation accuracy of MobileNetV3 increases slowly but eventually stabilizes at over 98%. The accuracy of DenseNet and ResNet-18 is slightly lower. The addition of the GARB improved the convergence speed and stability of the GA_ResNet model, enabling it to achieve high-precision convergence within fewer iterations.

**Figure 12:**
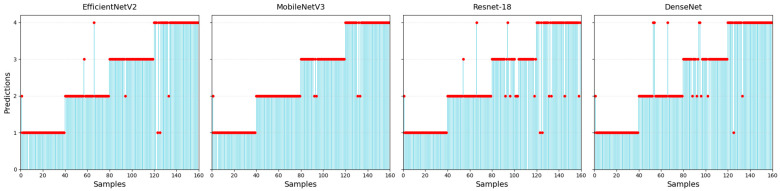
Predictions (the red dots) of different methods.

The prediction results of different methods are shown in Figure 12 and Table 4. All methods predict Sample 2 of Model 1 as Model 2. This indicates that the spectrum of Sample 2 is similar to Model 2’s. Only MobileNetV3-S predicts Sample 67 of Model 2 correctly, while it makes mistakes for four other samples. The metrics in Table 4 indicate that the GA-ResNet model achieves the highest values across all three metrics. The performance of the EfficientNetV2-S model is slightly lower than that of MobileNetV3-S, and although the DenseNet model improved its generalization ability through dense connections, it required the longest training time, and only achieved an accuracy of 92.50%. The ResNet-18 model yielded the lowest results in all three metrics, indicating insufficient capability in defect identification. Although the proposed GA-ResNet model does not have an obvious advantage in terms of training time, it improves the recognition accuracy of anchorage quality.

### 4.2. Ablation Study

**Figure 13:**
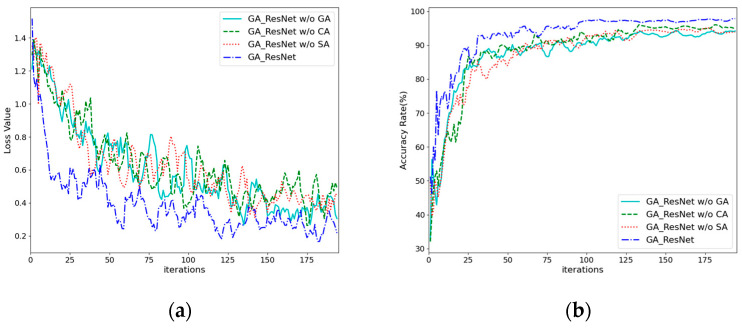
Training loss and validation accuracy curves of ablation study: (**a**) train losses; (**b**) validation accuracy.

In this section, we validate the effectiveness of the gated attention, channel attention, and spatial attention mechanisms. Three ablation studies were designed, resulting in the following: (1) the GA-ResNet model without gated attention (GA-ResNet w/o GA), in which the spatial and channel attention features are directly summed; (2) the GA-ResNet model without channel attention and gated attention (GA-ResNet w/o CA); and (3) the GA-ResNet model without spatial attention and gated attention (GA-ResNet w/o SA). The training process and results are shown in Figure 13. After removing some attention modules, the convergence speed of the training process slows down and the accuracy of the validation set decreases.

**Figure 14:**
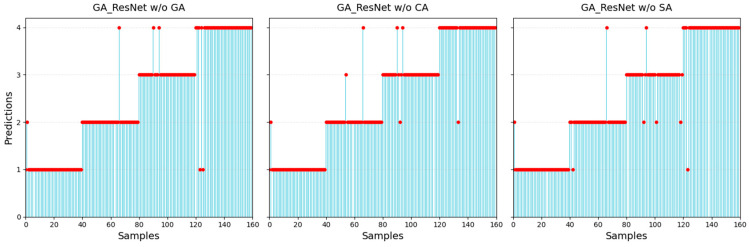
Predictions (the red dots) of ablation models.

The test results of the ablation experiments are shown in Figure 14 and Table 5. After removing the gated attention, the recognition accuracy decreases by approximately 2.5%. When channel attention or spatial attention is also removed, the recognition accuracy decreases by approximately 3.12% and 3.75%, respectively. In the ablation experiments, the changing trends of precision and F1 score are consistent with the trend of accuracy. The results indicate that after adding spatial or channel attention in resnet-18, the prediction accuracy of anchorage quality is similar to that of EfficientNetV2-S. When both types of attention were applied simultaneously, the accuracy slightly increased. After dynamically adjusting the weights of both with the gating mechanism, the metrics were further improved.

## 5. Conclusions

Anchorage defects can affect anchoring forces and, in severe cases, lead to anchorage failure. In this study, ultrasonic guided wave signals of anchor bolts with different anchorage qualities were tested, and time-domain and frequency-domain analyses were performed on these signals. Finally, time–frequency diagrams were selected as key features to explore the feasibility of using the GA-ResNet model to identify types of anchorage defects.

Based on the analysis of the bolt’s guided waves collected in the laboratory, it was shown that the staircase distortion and initial truncation time can affect the time-domain and frequency-domain distribution features. These issues introduce errors in the calculation of guided wave velocity and variations in frequency spectrum peaks.

We compared the prediction performance of the GA-ResNet with other classical models using the time–frequency spectrum as input. The results show that the GARB in GA-ResNet improves prediction accuracy. However, it should be noted that, for guided wave samples in extremely noisy environments, the model still cannot avoid prediction errors.

Our study has certain limitations: first, the variety of test models are relatively limited, and further research is needed on the behavior of guided waves in anchorage defects with different locations and lengths. In addition, the test data were collected in a laboratory environment, and the noise was artificially added. In actual support engineering, the guided waves of anchor bolts are disturbed by factors such as rock mass structure, grouting materials, the anchor bolt itself, and environmental noise, resulting in more complex guided wave signals, which require in-depth investigation in subsequent work.

## Figures and Tables

**Table 1:** Defect parameters of the four anchorage models.

Name	Defect Length L1/m	Defect Length L2/m	Location of Grouting Defect/m	Description
Model 1	-	-	-	No defect
Model 2	0.1	-	1.1	Single small defect
Model 3	0.1	0.1	0.3/1.1	Two small defects
Model 4	0.5	-	0.7	Single large defect

**Table 2:** Composition ratios of the concrete materials.

Materials	Sand	Cement	Water	Large Grave	Small Grave
Concrete 1	6.7	5.3	1.6	6.1	4.1
Concrete 2	4	2	1	-	-

**Table 3:** Arriving time and wave velocities of the four anchorage models.

Name	Received Guide Wave	First Wave Time/ms	Reflection Time from Front Face/ms	Reflection Time from Bottom End/ms	Velocity of Free Bolt/m·s^−1^	Velocity of Anchorage Section/m·s^−1^
Model 1	First	0.0801	0.2599	0.9799	5228.0	4166.7
	Second	0.1302	0.3100	1.0400	5228.0	4109.6
Model 2	First	0.0901	0.2599	0.9403	5535.9	4409.2
	Second	0.0901	0.2503	0.9403	5867.7	4347.8
Model 3	First	0.1507	0.3300	1.0604	5242.6	4107.3
	Second	0.0801	0.2604	0.9298	5213.5	4481.6
Model 4	First	0.1202	0.3099	0.9604	4955.2	4611.8
	Second	0.3204	0.5007	1.1702	5213.5	4480.9

**Table 4:** Recognition results of different methods.

Method	Accuracy Ratio	Precision Ratio	F1 Score
ResNet-18	90.00	91.04	90.09
DenseNet	92.50	92.98	92.53
MobileNetV3-S	96.88	97.22	96.93
EfficientNetV2-S	95.63	95.67	95.62
GA-ResNet	98.75	98.77	98.75

Note: results are presented as percentages (%).

**Table 5:** Recognition results of ablation study.

Method	Accuracy Ratio	Precision Ratio	F1 Score
GA-ResNet w/o GA	96.25	96.33	96.26
GA-ResNet w/o SA	95.00	95.18	95.00
GA-ResNet w/o CA	95.63	95.73	95.64
GA-ResNet	98.75	98.77	98.75

Note: results are presented as percentages (%).

## Data Availability

Data are available on request.
